# Is It so Severe for Social Entrepreneurship in a Transitional Economy? The Role of Work-Related Wellbeing and Political Connection in Shaping the Exit Intention

**DOI:** 10.3389/fpubh.2022.883153

**Published:** 2022-06-30

**Authors:** Jianing Dong, Xiao Wang, Xuanwei Cao, David Higgins

**Affiliations:** ^1^International Business School Suzhou, Xi'an Jiaotong-Liverpool University, Suzhou, China; ^2^Management School, University of Liverpool, Liverpool, United Kingdom

**Keywords:** social entrepreneur, entrepreneurial exit intention, prosocial motivation, transitional economy, work-related wellbeing, political connection

## Abstract

In the context of a transitional economy, there are much more studies with a heroic characterization of social entrepreneurs, whereas there is limited exploration of their less positive stories. A range of studies tried to address this issue, although very few delved into the “inner layer” (work-related mental health) to unveil the mechanism of how social entrepreneurs develop their intention to quit their businesses. With a sample of 196 social business owners from China, this research focuses on the prosocial motivation of social entrepreneurs as well as its impacts on their work-related wellbeing and thus their business exit intention. With the partial least squares structural equation modeling, this research finds that prosocial motivation decreased entrepreneurs' partial work-related wellbeing, increasing their exit intention, and the mediating effects among the three components of work-related wellbeing were different. Furthermore, this research finds that work-related wellbeing's impact on exit intention was largely stronger for the social entrepreneurs without political connections.

## Introduction

Exiting decision is an inevitable component of the entrepreneurial process and central to entrepreneurial decision-making research (1–4). When entrepreneurs are confronted with pressures, they will more or less decide whether to persist or pull the plug and exit the business (5–7). Nevertheless, very little research has attempted to document or investigate it (4, 8–10). This is particularly prominent in social entrepreneurship research, somewhat owing to the excessive heroic characterization of a social entrepreneur highlighting their success in improving people's lives, compared to the limited concerns on their less positive stories (11, 12). As it is much more common for an entrepreneur, especially a social entrepreneur, to cease his or her business, while the success is rare and hardly replicable, conducting in-depth research on the entrepreneurial failure becomes rather necessary and meaningful (12).

One of the research directions that arouses great interest in social entrepreneurial exit decision is how it occurs in the context of a transitional economy (12–14). Although we know that institutional environments imply constraints, incentives, and resources jointly affecting entrepreneurs and their activities (12, 15, 16), how exactly social entrepreneurship is affected is rarely empirically investigated (12–14). Moreover, scholars highlighted that current studies deficiently unveil how institutions interplay with social entrepreneurship generally and in non-US and non-Western institutional contexts (14, 17). Therefore, narrowing those gaps by investigating the exit of social entrepreneurs in the institutional context of a transitional economy can substantially contribute to the comprehensive understanding of social entrepreneurship.

As a psychological antecedent of entrepreneurial exit, the typical personality trait of social entrepreneurs—prosocial motivation, has attracted increasing research interest (12, 18). However, current research findings on the relationship between prosocial motivation and entrepreneurial exit are controversial. On the one hand, Mcmullen and Bergman (19), as well as Cardon and Wincent (20), suggested that the feelings of commitment toward their ventures (as “their babies”) evoked by prosocial motivation can impede their exit. On the other hand, Renko (20) as well as Wennberg, Wiklunc (21) indicated a contradictory view that social entrepreneurs are less likely to be successful in developing a viable firm than the entrepreneurs who are mainly motivated by financial goals, inevitably leading to their exit.

Addressing the debate above, scholars suggest introducing a mediator that can play an essential role in the relationship between prosocial motivation and exit intention (22, 23) for two reasons. First, it might be simplistic to investigate whether prosocial motivation will determine entrepreneurial exit; rather, with a zoomed-in lens, before taking the substantial step of ceasing a business, there can be both expediting and impeding intentions around such a step (24, 25). Thus, in-depth research is needed. Second, as failure is relatively common for entrepreneurship and even more for social entrepreneurship, due to the commitment to both economic and social value creation (26), exit intention is rather critical and hardly ignorable (19, 20). Previous research has indicated that work-related attitudes can be such a mediating variable between personality traits (in this research: prosocial motivation) and job-related outcomes (in this research: exit intention) (23), while work-related wellbeing essentially indicates a pervasive and persistent attitude (positive or negative) toward job or job situation (27–30).

In addition to the work-related wellbeing, in the context of a transitional economy, the impacts of response to and interaction with the environment can be hardly ignored as well. Although the transitional economy's institutional environment is unsupportive to social business (14, 27), several social enterprises have successfully emerged in such a context in China (14, 27). This somewhat challenges the predominant view on the relationship between institutional environment and entrepreneurial exit intention (28–30), assuming that the latter is uniformly impacted by the former. Nevertheless, firms' linkages with institutional authorities are diverse and heterogeneous (31, 32). This type of difference in political connections thus somewhat alters how entrepreneurs respond to the institutional environment of a transitional economy, which in turn, implies another research gap: how social entrepreneurs' divergent political connections affect their exit intention in the context of a transitional economy (12–14).

Accordingly, with a sample of 196 social entrepreneurs in China and the method of PLS-SEM (33) operated by SmartPLS (v.3.3.3), this research investigated how their prosocial motivation affects their exit intention mediated by work-related wellbeing (three dimensions: job satisfaction, work anxiety, and work burnout) in the context of a transitional economy, and how political connections can alter those impacts. The findings indicate that job satisfaction and work anxiety separately mediates prosocial motivation's effect on exit intention, while the mediating effects of work burnout is not significant. Moreover, we find that political connection moderates most of the relationships between work-related wellbeing and exit intention: job satisfaction and work anxiety's effects on exit intention are stronger for the social entrepreneurs without political connections than the ones with political connections, while the moderating effect of political connection on the relationship between work burnout and exit intention is insignificant.

The findings of this study imply three contributions. First, it furthers the researches on the relationship between prosocial motivation and exit intention (19, 20, 34) by unveiling the role of social entrepreneurs' work-related wellbeing and by extending our understanding on what types of work-related wellbeing influence their exit intention. Second, it discusses the necessity of involving political connection in further understanding of the relationship between prosocial motivation and work-related wellbeing as well as its effect on social entrepreneurs' exit intention in the context of a transitional economy. Third, it supplements the knowledge about how social entrepreneurs can increase their success rate in the context of a transitional economy, although this context can be rather different from and harsher than the one of a developed economy (35).

## Theoretical Background and Hypothesis Development

Allport (36) and Eysenck (37) suggested the hierarchical approach to personality provides a structural basis for integrating personality traits, situations, and behavioral intentions of individuals. One of the key assumptions of the hierarchical approach is that personality flows from higher to lower levels of the hierarchy, leading to behavioral intentions of individuals (36, 37). At the higher level of the hierarchy are the basic personality traits (38), while at the lower level are the surface traits, which are more specific and have a significant effect on behavioral intention.

Basic personality traits are an enduring disposition that originate from genetics and early learning history (38), while surface traits are an enduring disposition to behave in a specific context. Mowen and Spears (39) claim that a situation's potential requirement, such as the role demands of a job as a server in a restaurant, exerts pressures on people to shape a subjective pattern for behaving in such a situation.

Researchers including Licata, Mowen (40)Licata, Mowen (41), Brown, Tom (42) and Prentice and King (43) suggest that: basic personality traits and contextual elements jointly impact surface traits that eventually affect behavioral intentions of individuals.

Following the hierarchical approach to personality (36, 37), prosocial motivation, “the desire to benefit others or expend effort out of concern for others,” is regarded as a basic personality trait and represents “a person's 'affective lens' (remains constant over the time) on the world” (44–46). Work-related wellbeing, a pervasive and persistent attitude (positive or negative) toward one's job or job situation, is normally regarded as a type of surface personality traits, jointly developed by prosocial motivation and contextual features (in this research: transitional economy) (47–50).

Largely, a transitional economy was typically under central planning by the government and is now becoming market-oriented (40). It mostly adopts various types and levels of pro-market reforms to decentralize and limit the state's control in market, privatize property rights, reduce industry entry barriers and minimize governmental intervention in resource allocation (51). However, this transition cannot be achieved with one step; normally, it takes a long journey with various defects in fostering entrepreneurship (52, 53). For example, despite the gradualism of marketization in China, the delay in granting full rights to private entrepreneurs largely reflects ideological rigidity and institutional inertia against changes (54). As a result, the regulation systems are still weak and the political uncertainties surrounding businesses are relatively high (52, 53). Largely, in a transitional economy, social entrepreneurship can be hardly supported and facilitated, due to the survival-oriented or short-term culture, incomplete institutional arrangement for supportive resource allocation, and ambiguous policy and administrative procedures (15, 55, 56). Thus, their work-related wellbeing based on prosocial motivation is likely affected negatively. Indubitably, social entrepreneurs cannot be just impacted by the environment without any reaction and thus interaction with it (15, 56). This, in turn, may alter the degree of their work-related wellbeing's influence on their intention to exit social entrepreneurship.

Mostly, work-related wellbeing includes three dimensions: job satisfaction, work burnout, and work anxiety (57–65), indicating an attitude (positive or negative) to rank one's job or job situation (47–50). The three dimensions seem interrelated, but they can be independent of each other (66). For example, people may regard their work as difficult and demanding (low job satisfaction) and may suffer from performance anxiety (high anxiety), but still feel enthusiastic (low burnout) about their work (67). With the three dimensions of work-related wellbeing, we are able to disentangle the impact mechanism between prosocial motivation and exit intention via each of the dimensions.

### Prosocial Motivation, Job Satisfaction and Exit Intention

Job satisfaction is commonly defined as an attitudinal evaluative judgment of one's job or job experiences (68). In a transitional economy, prioritizing the values and beliefs of materialism can cause high levels of social injustice and disparity in wealth derived from the unjust social conditions, creating a society that is socially ill and ethically apathetic (69). The local opinion leaders, key stakeholders, or communities may form values, beliefs and hopes incongruent with the ones of entrepreneurs with prosocial motivation (70). Moreover, the distorted values and beliefs may weaken the formal institutions' efficacy and incubate a propensity for the public to be less concerned about the impact of ethical or responsible social behaviors without guilt (69). Under such a circumstance, social entrepreneurs' original intentions, values and implications can be hardly recognized and comprehended, furthering potential conflicts with the local opinion leaders, key stakeholders or communities. This can transform the entrepreneurs with prosocial motivation into a minority, impeding solution development for the social problems and eventually diminishing their job satisfaction (14, 71, 72). Grounded in the relationship between job satisfaction and exit intention, turnover theory suggests that a lower level of job satisfaction can cause a higher level of exit intention (73), as low-level job satisfaction implies that individuals will decrease their commitment to work and doubt their career choice. When one's job satisfaction deviates from his or her expectation, lower job satisfaction provides immediate aversive feedback to avoid pain from the work (29, 74, 75), resulting in low-level productivity and high-level absence and expediting higher exit intention (76, 77). Hence, prosocial motivation can incur extra burdens and pertinent pressures (78, 79), decreasing job satisfaction. And the weakened job satisfaction can undermine their job productivity and efficacy, as the social entrepreneurs may negatively interpret their works, and even start to doubt their work's values and social identity (29, 74, 75). To avoid a worse situation, they may choose latent escape and job absence, fostering their exit intention. Therefore, this research hypothesizes:

*Hypothesis1a: Prosocial motivation is negatively related to job satisfaction*.
*Hypothesis1b: Job satisfaction mediates the relationship between prosocial motivation and entrepreneurial exit intention*.

### Prosocial Motivation, Work Anxiety and Exit Intention

Work anxiety is defined as an emotional state of perceived apprehension and increased distress (80, 81), and characterized by worry and uneasiness about one's job performance (82). In a transitional economy, social ventures often face tensions related to scarcity of resources, especially financial resources (83, 84). For instance, the unaddressed issues about the ideological status of social enterprises in China can engender considerable uncertainties for decisions on policies such as tax exemptions and subsidies (85). This institutional ambiguity created by administrative inaction can undermine the critical legitimacy, support, and resources that can enhance social ventures' survival (86). For example, as there is no legal framework for social enterprises in a transitional economy mostly, financial institutions mostly do not lend money to this kind of organizations of which the priority is not profitability (87–90). However, given that adequate income and financial support is a buffer against anxiety and psychological strain of running a business (91), social entrepreneurs who suffer from income issues and scarcity of financial resources may develop anxiety since their strong commitment to a social business can be jeopardized (92–94). Meanwhile, work anxiety creates feelings of tension, potentially affecting the entire work process and even the outcome (95). Furthermore, this tension prompted by work anxiety can be converted into affective rumination (95), inducing escape from the work for the psychological restoration (95–97). Hence, when the work anxiety stemming from the contradiction between their strong commitment to social businesses and financial hardship increases, they may develop more feelings of tensions that can be transformed into stronger willingness to escape from their current works for the psychological restoration, increasing their intention to quit the social business. Therefore, this research hypothesizes:

*Hypothesis2a: Prosocial motivation is positively related to work anxiety*.
*Hypothesis2b: Work anxiety mediates the relationship between prosocial motivation and entrepreneurial exit intention*.

### Prosocial Motivation, Work Burnout and Exit Intention

Work burnout refers to the condition of physical and emotional exhaustion, as well as the associated negative attitudes resulting from the intensive interaction with the people at work (98). On the one hand, in a transitional economy, the non-supportive and unclear rules and regulations plus the fear of violating them increase the psychological burden of running a social enterprise (14). Besides, social entrepreneurs need to respond to relatively more governmental bureaucracy and political uncertainty in a transitional economy (27, 99, 100). This not only can impair the potential capacity to obtain resources to pursue both the economic and prosocial targets (3, 45), but also can create a tension between unsupportive and unclear regulations and entrepreneurial activities. On the other hand, through the process of social entrepreneurship in a transitional economy, social entrepreneurs are trying to stimulate a re-evaluation of the social values stemming from the institutions or non-institutions and retrieve the prosocial values (101). But attempts to alter the prevailing social values by introducing alternate values are often associated with confrontational approaches and tension between the alternate values and the dominant norms and values of communities and larger societies (88, 102). As a result, the tension between social entrepreneurship and non-supportive and unclear regulations, together with the tension between the alternate values and dominant norms and values, engender a burnout experience (103–105). Individuals who feel burnout at work are less likely to be satisfied and more likely to make a change (106), including work termination. Several studies have provided evidence that burnout is strongly associated with work withdrawal behavior. High levels of work burnout, which in turn, can be transformed into counterproductive behaviors (e.g., turnover, absenteeism, etc.). Hence, social entrepreneurs who feel burnout are more likely to become unsatisfied and counterproductive, which in turn, may induce withdrawal behaviors (107, 108) and even disengagement from the venture with consideration of leaving or exiting the social business entirely (109). Therefore, this research hypothesizes:

*Hypothesis3a: Prosocial motivation is positively related to work burnout*.
*Hypothesis3b: Work burnout mediates the relationship between prosocial motivation and entrepreneurial exit intention*.

### Entrepreneur's Political Connection as a Moderator

Prior studies claim that in transitional economies, social mechanisms (e.g., social networks, kinship networks) can be employed to buffer the negative effects on entrepreneurship caused by institutional deficiencies (28, 54, 110). Given the significant role of government and political authorities in transitional economies, political connections (as a social mechanism) are likely to be perceived as indispensable (27), potentially moderating the effects of social entrepreneurs' work-related wellbeing on their exit intention.

In a transitional economy that prioritizes materialism's values and beliefs, prosocial values and motivation can hardly be recognized by local opinion leaders, key stakeholders or communities, leading to lower job satisfaction and thus higher exit intention (70). But the political connections of social entrepreneurs may weaken the negative relationship between job satisfaction and exit intention. In transitional economies, local governments can be a critical source of information related to social entrepreneurial opportunities, and political connections can serve as informational cues to help identify such opportunities, drawing the social entrepreneurs' attention to the unaddressed social issues (111, 112), and thus confining their negative sense-making due to the impaired job satisfaction (29, 74, 75). Therefore, the negative effect of weakened job satisfaction on exit intention can be ameliorated.

In addition, previous research claims that in the context of an emerging economy, social entrepreneurs may develop anxiety because they need to respond to the lack of critical legitimacy, support, and resources resulting from the institutional ambiguity (86). Although such work anxiety can cause an increase in exit intention due to the tension between their commitment and the difficulties as well as their potential affective rumination (92–94), political connections may provide entrepreneurs a sense of security in a such a context (27). Given the incompletely developed market mechanism and resourceful government, political connections may help attain access to more information and details about the social entrepreneurs' peers or similar entrepreneurs and how they sustain their businesses (113, 114). Those complete or partial stories can inspire the social entrepreneurs with weakened work anxiety, encouraging them to learn from the stories (115, 116). This, in turn, can somewhat mitigate the tension and even affective rumination, thus alleviate the negative effect of work anxiety on exit intention.

Prior studies revealed that social entrepreneurs need to respond to considerable governmental bureaucracy and political uncertainty in a transitional economy (99, 100), stimulating work burnout (103–105) and subsequent entrepreneurial exit (107–109). But the political connections of social entrepreneurs may counteract this effect. Based on the reciprocity principle in political connections, both sides will have to benefit each other to sustain the relationship (99). In transitional economies, the government normally does not have sufficient resources to engage in social welfare projects (117), thus the reciprocity principle in political connections implies possible congruence and entanglement between the government and social enterprises in terms of “doing good” (99, 118). Accordingly, this reciprocal and continuous relationship can facilitate social entrepreneurs considering potential favorable actions of the government besides simply quitting their businesses, when they feel work burnout caused by the aforementioned bureaucracy and political uncertainty. This, in turn, can ameliorate the negative effect of work burnout on exit intention. Therefore, based on the arguments above, this research hypothesizes:

*Hypothesis4a: The relationship between job satisfaction and exit intention is stronger for social entrepreneurs without political connections*.
*Hypothesis4b: The relationship between work anxiety and exit intention is stronger for social entrepreneurs without political connections*.
*Hypothesis4c: The relationship between work burnout and exit intention is stronger for social entrepreneurs without political connections*.

**Figure 1:**
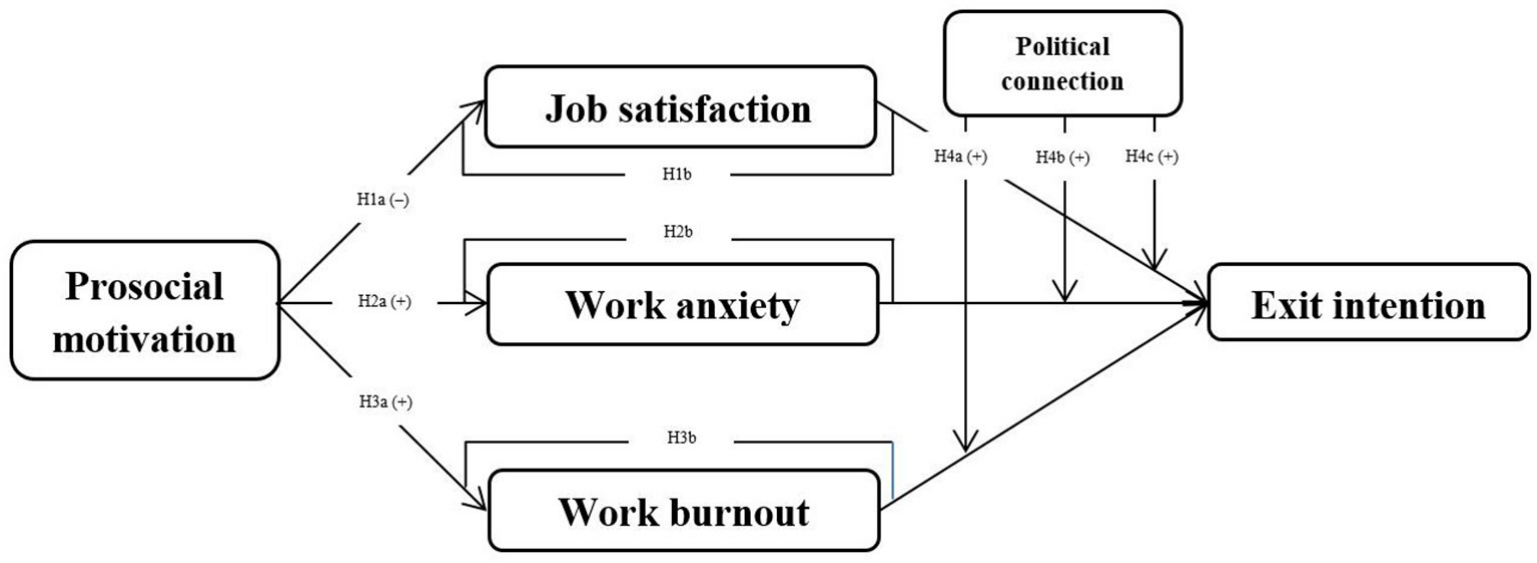
Theoretical model.

Figure 1 shows theoretical model.

## Method

### Sample and Procedure

Data for this research were collected in China, which is a typical context with the issues for social entrepreneurship raised above. We contacted the All-China Federation of Industry and Commerce for the data collection. This organization is a quasi-government organization of private firms that consists of business owners from firms of different sizes in various industries across China. It operates at the national, provincial, municipal and county level. The data were collected via an online questionnaire with Wenjuanxing (a survey tool), responded by the entrepreneurs who participated in the two large-scale colloquiums (onsite) organized by this organization in July (Jinan) and August (Qingdao) in 2021.

Since the questionnaire adopted by prior studies was initially developed in English, this research adopted the approach suggested by Brislin (119) for the translation. After the questionnaire draft was completed, a pilot test was performed (n = 50) to check whether it was necessary to make any adjustments. Finally, with a complementary literature review and field interviews, 22 items for seven constructs were eventually adopted. The Cronbach's alpha value of the pilot test was over 0.7, indicating that the internal consistency and stability of the questionnaire were acceptable (120).

We obtained 196 responses out of 450 invitations; the response rate is 43.6%. The questionnaire consisted of a general filter question and 7-point Likert items. According to the Global Entrepreneurship Monitor (GEM), the general filter was deployed to identify social entrepreneurs for this study:

“Are you, alone or with others, currently trying to start or currently owning and managing any kind of activity, organization or initiative that has a particularly social, environmental or community objective? This might include providing services or training to socially deprived or disabled persons, using profits for socially-oriented purposes, organizing self-help groups for community action, etc.”

Entrepreneurs marking “no” were identified as ordinary/commercial entrepreneurs and excluded from this research; while the entrepreneurs choosing “yes” were regarded as social entrepreneurs for this research (121). This method has been widely adopted by other studies on social entrepreneurship (15, 122).

After screening for the invalid samples with significant missing or apparently problematic values, the sample size of this research remained to be 196. Among the respondents, 82.2% were more than 35 years old; 55.6% were women; 69.9% were married or living with a partner; and 52.6% of them had a bachelor's degree. Table 1 shows an overview of the sample demographics.

**Table 1:** Sample demographics.

**Characteristics**	**Frequency**	**Percent (%)**
Age		
18–25	9	4.6%
26–35	26	13.3%
36–45	106	54.1%
46–55	55	28.1%
Gender		
Male	87	44.4%
Female	109	55.6%
Marital status		
Married	137	69.9%
Non-married	59	30.1%
Length of current business ownership		
<3 years	56	28.6%
3–5 years	62	31.6%
6–10 years	36	18.4%
11–15 years	29	14.8%
>15 years	13	6.6%
Educational level		
Junior high school	0	0%
High school or equal	5	0.31%
Junior college	37	18.9%
Bachelor degree	103	52.6%
Postgraduate or above	51	26.0%

### Variables and Measurement

#### Dependent Variable

##### Exit Intention

This research measured entrepreneurs' exit intention using the three items developed by Pollack, Vanepps (123). The entrepreneurs responded to each of them with a 7-point Likert scale ranging from 1 (strongly disagree) to 7 (strongly agree).

#### Independent Variable

##### Prosocial Motivation

This research measured the prosocial motivation with the four items adopted by Grant (124), and a 7-point Likert scale ranging from 1 (strongly disagree) to 7 (strongly agree) for each of the items.

#### Mediating Variable

##### Job Satisfaction

Based on the elaboration of the advantages (125) following prior studies (126, 127), this research measured entrepreneurs' job satisfaction with a single item developed by Chordiya, Sabharwal (127): “Generally speaking, I am satisfied with my job”, and a 7-point Liker scale ranging from 1 (strongly disagree) to 7 (strongly agree).

##### Work Anxiety

We measured work anxiety using the four-item general work anxiety scale developed by Haider, Fatima (128). Entrepreneurs responded to each of them with a 7-point Liker scale ranging from 1 (strongly disagree) to 7 (strongly agree).

#### Work Burnout

We adopted the ten-item general work burnout scale developed by Malach-Pines and Ayala (129). Entrepreneurs responded to each of them with a 7-point Likert scale ranging from 1 (strongly disagree) to 7 (strongly agree).

#### Moderating Variable

##### Political Connection

Following the representative studies (130, 131), affiliation with the state's political councils was employed by this study as an indicator of political connection. The survey asked whether the entrepreneur served as a representative in the National People's Congress (NPC) or Chinese People's Political Consultative Conference (CPPCC) at a national, provincial, municipal, or county level, since those two are the most important political institutions in which entrepreneurs have opportunities to develop political connection (132, 133). In this study, the respondents without political connection were coded as “1” and the respondents with political connection were coded as “2.”

A summary of the operational definitions is shown in Table 2. And the English questionnaire has been appended, presenting details of all the measurements (see Appendix A).

**Table 2:** Operational definition.

**Construct**	**Definition**	**Source**
Exit intention	An entrepreneur's desire or goal, at some point in the future, to leave his or her venture.	Pollack et al. (123)
Prosocial motivation	The desire to benefit others or expend effort out of concern for others.	Grant (124)
Job satisfaction	An attitudinal evaluative judgment of one's job or job experiences.	Chordiya et al. (127)
Work anxiety	An emotional state of perceived apprehension and increased arousal.	Haider et al. (128)
Work burnout	The condition of physical and emotional exhaustion.	Malach-Pines and Ayala (129)

### Measurement of Control Variables

In accordance with most of the entrepreneurship studies, we included several demographic variables as the control variables (see Table 1) due to their potential impacts on sustaining social entrepreneurship (134, 135), such as age (136), educational achievement (coded as “1” = “Junior high school,” “2” = “High school or equal,” “3” = “Junior college,” “4” = “Bachelor degree,” and “5” = “Postgraduate or above”), gender (137) and time length of current business ownership.

### Analytical Techniques

As an exploratory study, the partial least squares structural equation modeling (PLS-SEM) was adopted. This method is suitable for studying what has not been well tested before (138)—in this case, lack of knowledge or studies about the relationship between prosocial motivation and entrepreneurial exit intention. To decrease measurement error and avoid collinearity while examining the complicated relationship between prosocial motivation, job satisfaction, work burnout, work anxiety and exit intention, PLS becomes more suitable for this research than other SEM methods (139).

## Results

Deploying PLS-SEM, this research followed the two-step approach (140): the first step is to assess the outer model and the second step is to examine the inner model. Table 3 presents the correlations and descriptive statistics for the constructs included in the research.

**Table 3:** Descriptive statistics and correlations.

**Variable**	**Mean**	**S.D**.	**PM**	**JS**	**WA**	**WB**	**EI**
PM	4.91	0.78	NA				
JS	1.78	0.74	−0.33^**^	NA			
WA	4.98	1.04	0.52^**^	−0.11	NA		
WB	3.82	1.37	−0.09	0.43	0.12	NA	
EI	5.12	1.03	0.52^**^	−0.56^**^	0.41^**^	−0.04	NA

*PM, prosocial motivation; JS, job satisfaction; WB, work burnout; WA, work anxiety; EI, exit intention*.

** *p < 0.01*.

### Outer Model and Scale Validation

The related tests for the outer model included the reliability of each item as well as the internal consistency, convergent validity, and discriminant validity of each construct. For the reliability of each item, the threshold value should be 0.5 for the individual reliability (141), and Fornell and Larcker (142) suggested the Cronbach's alpha value should be 0.7 for statistical significance. Besides, Fornell and Larcker (142) recommend a value 0.7 for the composite reliability, while Fornell and Larcker (142) recommend a value that is 0.5 for the AVE to evaluate the convergent validity of each composite. As Table 4 shows, all the factor loadings are above 0.5, the Cronbach's alpha value is >0.7, the values for the composite reliability are above 0.7 (adequate internal consistency), and the AVE values are all above 0.5 (good convergent validity).

**Table 4:** Reliability and AVE of the outer model.

**Construct**	**Indicators**	**Cronbach's**	**Factor**	**Composite**	**AVE**
		**alpha**	**loading**	**reliability**	
PM	PM 1	0.847	0.869	0.897	0.686
	PM 2	–	0.845	–	–
	PM 3	–	0.763	–	–
	PM 4	–	0.831	–	–
WB	WB 1	0.968	0.625	0.953	0.677
	WB 2	–	0.581	–	–
	WB 3	–	0.677	–	–
	WB 4	–	0.766	–	–
	WB 5	–	0.911	–	–
	WB 6	–	0.946	–	–
	WB 7	–	0.840	–	–
	WB 8	–	0.944	–	–
	WB 9	–	0.946	–	–
	WB 10	–	0.885	–	–
WA	WA 1	0.925	0.885	0.945	0.812
	WA 2	–	0.932	–	–
	WA 3	–	0.904	–	–
	WA 4	–	0.887	–	–
EI	EI 1	0.931	0.910	0.956	0.880
	EI 2	–	0.962	–	–
	EI 3	–	0.941	–	–

*PM, prosocial motivation; WB, work burnout; WA, work anxiety; EI, exit intention*.

*Job satisfaction is a single–item construct*.

Discriminant validity can be analyzed by checking that the correlation between each pair of constructs is not greater than the value of the square root of the AVE for each construct and by using the heterotrait-monotrait ratio (HTMT). Normally, the HTMT threshold for acceptable discriminant validity is 0.90 (143): if the HTMT value is below 0.90, the discriminant validity is acceptable, which is as Table 5 shows. Moreover, as Table 6 shows, the comparison of cross loadings and factor loadings for each indicator signifies reasonable discriminant validity, since the factor loading of each scale item for its assigned latent construct is higher than its loading on any other constructs (141). Therefore, the constructs in this research have good discriminant validity.

**Table 5:** Discriminant validity results – HTMT.

**Factors**	**EI**	**JS**	**PM**	**WA**	**WB**
EI					
JS	0.578				
PM	0.589	0.357			
WA	0.445	0.112	0.586		
WB	0.054	0.048	0.161	0.125	

*PM, prosocial motivation; JS, job satisfaction; WB, work burnout; WA, work anxiety; EI, exit intention*.

**Table 6:** Standardized factor loadings and cross loadings of the outer model.

	**EI**	**JS**	**PM**	**WA**	**WB**
EI1	0.910	−0.571	0.469	0.335	−0.107
EI2	0.962	−0.532	0.498	0.422	−0.078
EI3	0.941	−0.466	0.503	0.479	−0.064
JS1	−0.558	1.000	−0.328	−0.113	0.055
PM1	0.440	−0.256	0.869	0.433	−0.312
PM2	0.376	−0.243	0.848	0.494	−0.080
PM3	0.455	−0.294	0.763	0.393	−0.090
PM4	0.461	−0.296	0.831	0.449	−0.240
WA1	0.255	−0.058	0.404	0.885	0.073
WA2	0.374	−0.130	0.469	0.932	0.003
WA3	0.312	−0.087	0.425	0.904	0.059
WA4	0.550	−0.116	0.575	0.884	0.039
WB1	−0.004	−0.015	0.012	0.082	0.624
WB10	−0.031	0.050	−0.130	0.129	0.885
WB2	0.009	0.062	0.102	0.163	0.581
WB3	0.049	0.019	0.067	0.207	0.677
WB4	0.001	0.011	−0.043	0.107	0.766
WB5	−0.067	−0.002	−0.151	0.032	0.911
WB6	−0.075	0.072	−0.116	0.086	0.946
WB7	−0.029	0.052	−0.103	0.121	0.840
WB8	−0.070	0.060	−0.182	0.078	0.944
WB9	−0.101	0.073	−0.246	0.019	0.948

*PM, prosocial motivation; JS, job satisfaction; WB, work burnout; WA, work anxiety; EI exit intention. The gray cells are the factor loadings of scale items for each construct*.

### Inner Model and Hypotheses Testing

**Figure 2:**
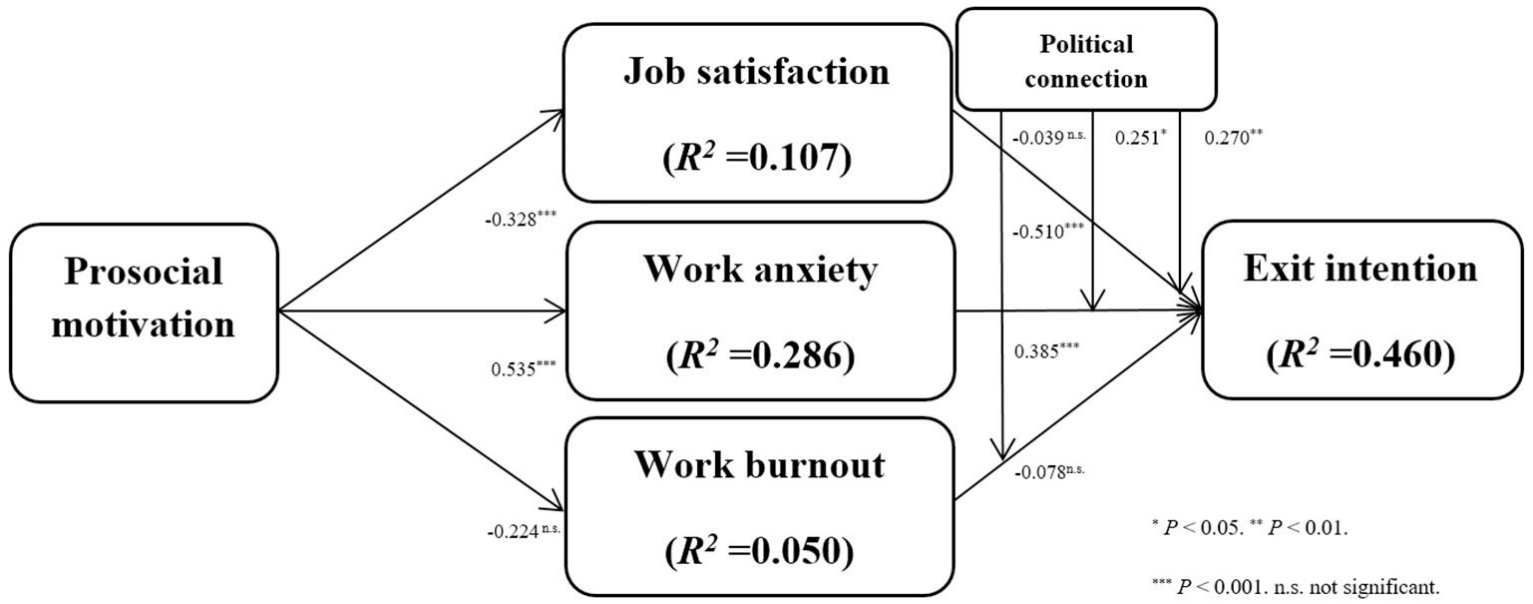
Path coefficient and R-squares of their inner model.

Figure 2 and Table 7 summarize the structural model from PLS analysis by showing the standardized path coefficients (β) and their significance (*t*-values) as well as the explained variance of endogenous variables (R^2^). We calculated *t*-values through a bootstrap approach based on 5,000 random resamples.

**Table 7:** Summary of inner model results.

**Hypotheses**	**Path coefficients (β)**	**t–value**	**Supported**
H1a: PM–>JS	−0.328^***^	5.382	Yes
H2a: PM–>WA	0.535^***^	8.358	Yes
H3a: PM–>WB	−0.224^n.s.^	1.248	No

*PM, prosocial motivation; JS, job satisfaction; WB, work burnout; WA, work anxiety*.

*** *P-value < 0.001;*

*n.s. not significant*.

*Number of bootstrap samples = 5,000*.

Figure 2 and Table 7 show that the prosocial motivation negatively and significantly affects job satisfaction, supporting hypothesis 1a (PM → JS: β = −0.328, *t*-value = 5.382); prosocial motivation positively and significantly impacts work anxiety, supporting hypotheses 2a (PM → WA: β = 0.535, t-value = 8.358); prosocial motivation has an insignificant impact on work burnout, rejecting hypotheses 3a (PM → WB: β = −0.224, *t*-value = 1.248).

Besides examining R^2^, we also tested the model's predictive validity by analyzing the predictive relevance of the exogenous variables Q^2^ (144). With regard to Q^2^, we find that the values of Q-square for job satisfaction (Q^2^ = 0.105), work anxiety (Q^2^ = 0.216), work burnout (Q^2^ = 0.001), and exit intention (Q^2^ = 0.397) are all larger than zero, suggesting that the theoretical model of this study has sufficient explanatory power.

### Testing of Mediation Effects

This study has three mediating variables, namely job satisfaction, work anxiety, and work burnout. This research conducted the Sobel z test and found that the Sobel test z of the two mediating variables (job satisfaction and work anxiety) were both above 1.96, which means that both two mediating variables had an intermediary effect, whereas work burnout did not have an intermediary effect (see Table 8) (145).

**Table 8:** Test of mediation effect.

	**Original**	**Standard Error**	**T Statistics**	
	**Sample (O)**	**(STERR)**	**(|O/STERR|)**	
PM –> JS	−0.329	0.090	3.647	
JS –> EI	−0.452	0.073	6.188	
PM –> WA	0.534	0.089	5.998	
WA –> EI	0.268	0.125	2.151	
PM –> WB	−0.223	0.213	1.050	
WB –> EI	−0.025	0.082	0.305	
PM –> EI	0.227	0.109	2.072	
	**PM–>JS–>EI**	**PM–>WA–>EI**	**PM–>WB–>EI**	**Total indirect effect**
Indirect effect	0.149	0.143	0.006	0.524
Sobel Z Test	3.14	2.02	0.29	–
VAF	0.284	0.273	0.011	0.567

*PM, prosocial motivation; JS, job satisfaction; WB, work burnout; WA, work anxiety; EI, exit intention*.

*Number of bootstrap samples = 5,000*.

Variance accounted for (VAF) refers to the proportion of indirect effects to total effects. In Table 8, The VAFs of the three mediating variables are 28.4, 27.3, and 1.1% respectively, which means that the total indirect effect explains 28.4, 27.3, and 1.1% of the total effect respectively. According to Hair Jr, Hult (141), if VAF > 80%, it is full mediation; if VAF ≤ 80%, it is partial mediation; if VAF < 20%, there is no mediation. Table 6 indicates that job satisfaction and work anxiety were significant partial mediators between prosocial motivation and exit intention, whereas work burnout was not a significant mediator. Hence, these results confirm hypotheses 1b and 2b while reject hypothesis 3b.

### Multi-Group Analysis

In this research, given political connection is a categorical variable (1 = without political connection, and 2 = with political connection), multiple group analysis procedure (PLS-MGA) via SmartPLS (Version 3.3.3) for group comparisons became an appropriate approach for the analysis. PLS-MGA was conducted with a bootstrapped sample of 5,000 to examine the statistical significance of the two comparable groups' path coefficients (146). The path coefficients of different groups allow us to see which path is distinct, how different the paths are, and whether there is difference in path direction. The results are presented in Table 9.

**Table 9:** Multi–group analysis results.

**Path**	**Pooled**		**Group A (Without PC)**		**Group B (With PC)**		**Grp A VS**	**Supported**
	***N*** **= 196**		***N*** **= 121**		***N*** **= 75**		**Grp B**	
	**β**	**CI**	**β**	**CI**	**β**	**CI**	***P*** **–value**	
JS–>EI	−0.510	(−0.608, −0.403)	−0.651	(−0.527,−0.229)	−0.382	(−0.760,−0.535)	0.008	YES
WA–>EI	0.385	(0.275, 0.507)	0.486	(0.352, 0.618)	0.234	(0.289,0.385)	0.030	YES
WB–>EI	−0.078	(−0.183, 0.099)	−0.066	(−0.252, 0.047)	−0.105	(−0.378,-0.197)	0.846	NO

*PM, prosocial motivation; JS, job satisfaction; WB, work burnout; WA, work anxiety; EI, exit intention; PC, political connection*.

*β, path coefficient; CI = 95% Confidence interval*.

The path coefficients (β) have been estimated, and the differences of the two coefficients have been analyzed. The results indicated that the path coefficient between prosocial motivation and job satisfaction for group A (without political connection) was significantly greater than that for group B (with political connection) (H4a: βdiff = 0.269, *p* = 0.008). Meanwhile, the path coefficient between prosocial motivation and work anxiety for group A (without political connection) was significantly larger than that for group B (with political connection) (H4b: βdiff = 0.252, *p* = 0.030). Therefore, H4a and H4b are supported.

Comparatively, the PLS-MGA results indicate that there was no statistically significant difference between the sub-sample of social entrepreneurs without political connection and the one with political connection in the path between prosocial motivation and work burnout. Accordingly, H4c is not supported.

## Discussion

Echoing the prior studies calling for in-depth investigation on the negative facets of social entrepreneurship (12, 147), this research unveiled how entrepreneurs' prosocial motivation can affect their exit intention in the context of a transitional economy through the mediation of work-related wellbeing. In addition to the theoretical implications, the findings have significant implications for the social entrepreneurs, especially those running social businesses in a transitional economy (12–14). By doing so, we shift the focus of prior research (3, 18) from the “bright side” to the “dark side” of its (prosocial motivation's) potential effect on entrepreneurs (45).

### Theoretical Implication

This study found support for the negative relationship between prosocial motivation and job satisfaction (H1a). This is not in line with the result reported by Brieger et al. (58) who found that entrepreneurs' prosocial characteristics positively impact job satisfaction in Germany. This difference is related to the developmental stage of an economy, and entrepreneurial activities are unquestionably embedded in the pertinent social and cultural norms and values (55). Compared to a developed economy like Germany, in a transitional economy, materialistic values and beliefs, instead of pursuing a balance between economic and social performance, are prioritized (58). Thus, social value creation embedded in entrepreneurial activities can hardly gain the respect of family members, friends and the broader community in a transitional economy, negatively affecting the social entrepreneurs' job satisfaction. Besides, previous research based on World Values Survey (WVS) (148) claimed a considerable variation in social entrepreneurial prevalence among different societal cultures. In a traditional or survival society, human beings' physical and economic security is regarded to have more priority over other issues, implying negative attitudes toward social entrepreneurial activities. Therefore, this research somewhat responds to prior studies calling for examining how different contextual conditions of diverse economies affect social entrepreneurial activities.

In addition, we found that prosocial motivation is positively related to work anxiety (H2a), which is unique since no prior studies have specifically investigated such a nexus between prosocial motivation and work anxiety. While Azmat, Ferdous (83) and Mair and Marti (84) suggested that social ventures often face tensions owing to scarcity of resources especially financial ones, our findings imply that entrepreneurs with strong prosocial motivation can be regarded as non-profit-driven in a transitional economy, leading to more difficulty of financing their enterprises and thus their work anxiety. Hence, this study somewhat extends the work of Azmat, Ferdous (83) and Mair and Marti (84).

Moreover, we found that prosocial motivation is indirectly related to exit intention via job satisfaction (H1b) and work anxiety (H2b) in a transitional economy. On the one hand, this finding echoes prior studies demonstrating how (dis)satisfaction with specific life domains (work, family) is linked to exit intentions (149–151). On the other hand, this corresponds to the call for studying potential mediating variables to enhance understanding of the connections between prosocial motivation and entrepreneurial exit intention (22, 152), advancing our relevant understanding (12, 58, 105).

Furthermore, we found that the relationship between job satisfaction and exit intention as well as work anxiety and exit intention is stronger for the social entrepreneurs without political connections, respectively (H4a and H4b). This implies that in spite of the negative work-related wellbeing of social entrepreneurs in a transitional economy caused by the unfavorable socioeconomic environment (13), connections with political authorities can provide buffers against its negative effect on their exit intention. For instance, the political connections can provide more information regarding potential societal issues and thus necessity of social works (27, 89), mitigating the negative effect of weakened job satisfaction on exit intention. Likewise, the political connections can help transfer the information about how an exemplary social entrepreneur in such a context managed to sustain his or her social venture (27, 130, 153), ameliorating the negative effect of attenuated work anxiety on exit intention. Hence, those findings are remarkable since it further implies the necessity of studies in the context of a transitional economy and the significance of “human condition,” which prevalent social entrepreneurship theories do not adequately include (12). As social entrepreneurs cannot be utterly reactive in the context of a transitional economy with more turbulent dynamics (14, 27), there can be more additional alternatives like political connections adopted by the social entrepreneurs, diminishing the exit intention. Furthermore, there could be a more complex mechanism leading to the exit intention. For instance, political connections might incur the reciprocity irrelevant to the growth of social entrepreneurship (99, 118), which in turn may further attenuate the weakened job satisfaction or work anxiety, somewhat undermining the buffering effect of political connections on the negative relationship between job satisfaction or work anxiety and exit intention. Therefore, those findings are remarkable also in terms of the implication for further studies, narrowing an essential gap in extant social entrepreneurship literature: comprehensive mechanisms that map how individual-level political connections aggregate into the entrepreneurial decision of social entrepreneurs (12, 147).

However, contrary to our prediction, we did not find support for prosocial motivation's effects on entrepreneurial work burnout (H3a), work burnout's mediating role in the relation between prosocial motivation and exit intention (H3b), and political connection's moderating effect on the nexus between work burnout and exit intention (H4c). The inconsistency between our prediction and the empirical results is probably due to the sampled entrepreneurs' age, gender, and marital status. First, over 70% of the respondents were below 45 years old in this research. Prior literature pointed out a non-linear trend of entrepreneurial enthusiasm: it escalates with age increase, peaking around the age of 35–44 (154). Accordingly, young, especially nascent entrepreneurs are more enthusiastic about starting an autonomous career and managing a business. Second, gender role theory claimed that most occupations remain gender-typed (155), and men in female-typed occupations reported more significant psychological distress and poorer self-evaluated health, and vice versa. Previous research claimed that social entrepreneurs are female-typed occupations (156, 157), while over 55% of the respondents were female in this research. Third, nearly 70% of the respondents were married in this research, and the spouse or partner can provide significant help to cope with the work burnout (158, 159). Therefore, the entrepreneurs' work burnout in this research might be less perceptible and underestimated.

### Practical Implication

According to this study, social entrepreneurs in a transitional economy tend to have a lower level of work-related wellbeing, escalating their exit intention that undermines their business sustainability and career development. Based on the findings, first, social entrepreneurs need to be fully aware of the role of work-related wellbeing in such a context, which may otherwise expedite entrepreneurial failure eventually. Second, given the critical role of work-related wellbeing in shaping the social entrepreneurs' exit intention, entrepreneurship educators may need to provide more knowledge and tools to enhance and maintain the social entrepreneurs' work-related wellbeing. Only focusing on the successful case studies for the training programs on entrepreneurship can be problematic and misleading. Third, relevant governmental agencies should provide more support such as relevant policies, facilities, training, and consultation to improve social entrepreneurs' work-related wellbeing. Fourth, establishing political connections according to relevant laws, regulations and policies with the governmental agencies or agents who support or need to support social businesses can be an alternative for social entrepreneurs surviving in the context of a transitional economy.

### Limitations and Future Research Directions

Before concluding, the limitations of this research should be noted. First, as the data of this study were collected in China which has a distinctive institutional and cultural environment, future research that replicates our findings in other distinctive institutional and cultural environments may strengthen the generalizability of our conclusions. Second, a sample without the imbalanced ratio of gender, marital status and educational level can be employed in future studies to test our findings and the potential moderating effects of gender, marital status and educational level can also be examined. Third, this research did not measure the respondents' actual exit. Although research on intentions indicates a high probability of pertinent action (particularly when individuals have perceived control over their actions), and 70% of those who had exit intention take the substantial step eventually (160), it is certainly plausible that the actual exit differ from the exit intention, and further studies could employ behavioral measurements to corroborate our findings. Finally, although this study supplements the yet rare quantitative studies in social entrepreneurship research (161), the detailed mechanism and the interplay between the variables are still unknown, which in turn may need qualitative approaches for more in-depth exploration.

## Data Availability Statement

The original contributions presented in the study are included in the article/supplementary materials, further inquiries can be directed to the corresponding author/s.

## Ethics Statement

Ethical review and approval was not required for the study on human participants in accordance with the local legislation and institutional requirements. The patients/participants provided their written informed consent to participate in this study.

## Author Contributions

Conceptualization: JD, XW, XC, and DH. Methodology, software, validation, formal analysis, investigation, resources, data curation, writing—original draft preparation, and visualization: JD. Writing—review and editing, supervision, and funding acquisition: XW. All authors contributed to the article and approved the submitted version.

## Funding

This work was supported in part by the XJTLU Doctoral Scholarship PGRS 1901010.

## Conflict of Interest

The authors declare that the research was conducted in the absence of any commercial or financial relationships that could be construed as a potential conflict of interest.

## Publisher's Note

All claims expressed in this article are solely those of the authors and do not necessarily represent those of their affiliated organizations, or those of the publisher, the editors and the reviewers. Any product that may be evaluated in this article, or claim that may be made by its manufacturer, is not guaranteed or endorsed by the publisher.

## Appendix

**Appendix A:** The questionnaire.

**Construct**	**Items**
Prosocial motivation (PM)	(1) I care about benefiting others through my work. (2) I want to have positive impact on others. (3) Because I want to have positive impact on others. (4) It is important to me to do good for others through my work.
Job satisfaction (JS)	(1) Generally speaking, I am satisfied with my job.
Work anxiety (WA)	(1) I have felt fidgety or nervous as a result of my job. (2) My job gets to me more than it should. (3) There are lots of times when my job drives me right up the wall. (4) Sometimes when I think about my job, I get a tight feeling in my chest.
Work burnout (WB)	When you think about your work overall, how often do you feel the following? (1) Tired (2) Disappointed with people (3) Hopeless (4) Trapped (5) Helpless (6) Depressed (7) Physically weak/Sickly (8) Worthless/Like a failure (9) Difficulties sleeping (10) “I've had it”
Exit intention (EI)	Participants rated the extent to which they would, in the next year? (1) Avoid entrepreneurial positions (2) Feel anxious about entrepreneurial positions (3) Feel less excited about entrepreneurial positions

## References

[1] Detienne DR. Entrepreneurial exit as a critical component of the entrepreneurial process: theoretical development. J Bus Ventur. (2010) 25:203–15. 10.1016/j.jbusvent.2008.05.004

[2] Shepherd DA Patzelt H. Harsh evaluations of entrepreneurs who fail: the role of sexual orientation, use of environmentally friendly technologies, and observers' perspective taking. J Manag Stud. (2015) 52:253–84. 10.1111/joms.12103

[3] Shepherd DA. Party On! a call for entrepreneurship research that is more interactive, activity based, cognitively hot, compassionate, and prosocial. J Bus Ventur. (2015) 30:489–507. 10.1016/j.jbusvent.2015.02.001

[4] Cefis E Bettinelli C Coad A Marsili O. Understanding firm exit: a systematic literature review. Small Bus Econ. (2021). 10.1007/s11187-021-00480-x

[5] Khelil N. The many faces of entrepreneurial failure: insights from an empirical taxonomy. J Bus Ventur. (2016) 31:72–94. 10.1016/j.jbusvent.2015.08.001

[6] Williamson AJ Gish JJ Stephan U. Let's focus on solutions to entrepreneurial ill-being! recovery interventions to enhance entrepreneurial well-being. Entrep Theory Pract. (2021) 45:1307–38. 10.1177/10422587211006431

[7] Stephan U Rauch A Hatak I. Happy Entrepreneurs? Everywhere? a meta-analysis of entrepreneurship and wellbeing. Entrep Theory Pract. (2022) 0:10422587211072799. 10.1177/10422587211072799

[8] Stephan U. Entrepreneurs' mental health and well-being: a review and research agenda. Acad Manag Perspect. (2018) 32:290–322. 10.5465/amp.2017.0001

[9] DeTienne DR Wennberg K. Research Handbook of Entrepreneurial Exit. Cheltenham: Edward Elgar Publishing (2015). 10.4337/9781782546979

[10] Jayawarna D Marlow S Swail J. A gendered life course explanation of the exit decision in the context of household dynamics. Entrep Theory and Pract. (2021) 45:1394–430. 10.1177/1042258720940123

[11] Kimmitt J Muñoz P. Sensemaking the ’Social'in social entrepreneurship. Int Small Bus J. (2018) 36:859–86. 10.1177/026624261878923034131355

[12] Tina S Foss NJ Stefan L. Social entrepreneurship research: past achievements and future promises. J Manage. (2018) 45:70–95.

[13] Sengupta S Sahay A Croce F. Conceptualizing social entrepreneurship in the context of emerging economies: an integrative review of past research from Briics. Int Entrepreneurship Manag J. (2018) 14:771–803. 10.1007/s11365-017-0483-2

[14] Bhatt B Qureshi I Riaz S. Social entrepreneurship in non-munificent institutional environments and implications for institutional work: insights from China. J Bus Ethics. (2019) 154:1–26. 10.1007/s10551-017-3451-4

[15] Stephan U Uhlaner LM Stride C. Institutions and social entrepreneurship: the role of institutional voids, institutional support, and institutional configurations. J Int Bus Stud. (2015) 46:308–31. 10.1057/jibs.2014.38

[16] Lee CK Simmons SA Amezcua A Lee JY Lumpkin G. Moderating effects of informal institutions on social entrepreneurship activity. J Soc Entrepreneurship. (2020):1–26. 10.1080/19420676.2020.1782972

[17] Tsui AS. Contributing to global management knowledge: a case for high quality indigenous research. Asia Pac J Manag. (2004) 21:491–513. 10.1023/B:APJM.0000048715.35108.a7

[18] Miller TL Grimes MG Mcmullen JS Vogus TJ. Venturing for others with heart and head: how compassion encourages social entrepreneurship. Acad Manag Rev. (2012) 37:616–40. 10.5465/amr.2010.0456

[19] Mcmullen JS Bergman B. Social entrepreneurship and the development paradox of prosocial motivation: a cautionary tale. Strateg Entrepreneurship J. (2017). 10.1002/sej.1263

[20] Renko M. Early challenges of nascent social entrepreneurs. Entrep Theory Pract. (2013) 37. 10.1111/j.1540-6520.2012.00522.x31094539

[21] Wennberg K Wiklunc J Detienne DR Cardon MS. Reconceptualizing entrepreneurial exit: divergent exit routes and their drivers. J Bus Ventur. (2010) 25:361–75. 10.1016/j.jbusvent.2009.01.001

[22] Rauch A Frese M. Let's Put the Person Back into Entrepreneurship Research: A Meta-Analysis on the Relationship between Business Owners' Personality Traits, Business Creation, and Success: European Journal of Work and Organizational Psychology: Vol 16, No 4. Eur J Work Organ Psychol. (2007). 10.1080/13594320701595438

[23] Lindblom A Lindblom T Wechtler H. Dispositional Optimism, Entrepreneurial Success and Exit Intentions: The Mediating Effects of Life Satisfaction. J Bus Res. (2020) 120:230–40. 10.1016/j.jbusres.2020.08.012

[24] Lortie J. For the Greater Good: Why and How Social Entrepreneurs Exit Social Ventures. In: Detienne D Wennberg K editors. Research Handbook of Entrepreneurial Exit. Cheltenham: Edward Elgar Publishing (2015). 10.5465/ambpp.2014.16704abstract

[25] Brigham KH Castro JD Shepherd DA. A person-organization fit model of owner-managers' cognitive style and organizational demands. Entrep Theory Pract. (2010) 31:29–51. 10.1111/j.1540-6520.2007.00162.x

[26] Zahra SA Gedajlovic E Neubaum DO Shulman JM. A Typology of social entrepreneurs: motives, search processes and ethical challenges. J Bus Ventur. (2009) 24:519–32. 10.1016/j.jbusvent.2008.04.007

[27] Ge J Stanley LJ Eddleston K Kellermanns FW. Institutional deterioration and entrepreneurial investment: the role of political connections. J Bus Ventur. (2017) 32:405–19. 10.1016/j.jbusvent.2017.04.002

[28] Estrin S Korosteleva J Mickiewicz T. Which institutions encourage entrepreneurial growth aspirations? J Bus Ventur. (2013) 28:564–80. 10.1016/j.jbusvent.2012.05.0019387815

[29] DeTienne D Wennberg K. studying exit from entrepreneurship: new directions and insights. Int Small Bus J. (2016) 34:151–6. 10.1177/0266242615601202

[30] Wennberg K Detienne DR. What Do We Really Mean When We Talk About 'Exit'? A critical review of research on entrepreneurial exit social science electronic publishing. Int Small Bus J. (2014) 32:4–16. 10.1177/0266242613517126

[31] Scott WR. Institutions and Organizations: Ideas. In: Interests, and Identities. 3rd edition. Los Angeles, CA: Sage Publications (2014).

[32] Powell WW DiMaggio PJ. The New Institutionalism in Organizational Analysis: Chicago, IL; London: University of Chicago press (2012).

[33] Ringle CM Wende S Becker J-M. Smartpls 3. Smartpls Gmbh, Boenningstedt. J Serv Sci Manag. (2015) 10.

[34] Grant AM. Relational job design and the motivation to make a prosocial difference. Acad Manag Rev. (2007) 32:393–417. 10.5465/amr.2007.24351328

[35] Courpasson D. On the Erosion of ’Passionate Scholarship'. London: Sage Publications Sage (2013). 10.1177/0170840613502292

[36] Allport GW. Pattern and Growth in Personality. (1961).

[37] Eysenck HJ. Dimensions of Personality. In: Strelau J Angleitner A editors. Explorations in Temperament: International Perspectives on Theory and Measurement. Boston, MA: Springer US (1991). p. 87–103.

[38] Mowen John C. The 3m Model of Motivation and Personality. New York, NY: Springer (2000 10.1007/978-1-4757-6708-7

[39] Mowen JC Spears N. Understanding compulsive buying among college students: a hierarchical approach. J Consum Psychol. (1999) 8:407–30. 10.1207/s15327663jcp0804_03

[40] Kriauciunas A Kale P. The impact of socialist imprinting and search on resource change: a study of firms in Lithuania. Strateg Manag J. (2006) 27:659–79. 10.1002/smj.537

[41] Licata JW Mowen JC Harris EG Brown TJ. On the trait antecedents and outcomes of service worker job resourcefulness: a hierarchical model approach. Acade Market Sci Rev. (2003) 31:256–71. 10.1177/0092070303031003004

[42] Brown Tom Mowen John. The Customer Orientation of Service Workers: Personality Trait Determinants and Influences on Self and Supervisor Performance Ratings. J Mark Res. (2002) 39:110–9. 10.1509/jmkr.39.1.110.18928

[43] Prentice C King BEM. Impacts of personality, emotional intelligence and adaptiveness on service performance of casino hosts: a hierarchical approach. J Bus Res. (2013) 66:1637–43. 10.1016/j.jbusres.2012.12.009

[44] Eisenberg NancyGuthrie Ivanna Cumberland K AmandaMurphy Bridget. Prosocial development in early adulthood: a longitudinal study. J Pers Soc Psychol. (2002) 82:993–1006. 10.1037/0022-3514.82.6.993 12051585

[45] Bolino MC Grant AM. The bright side of being prosocial at work, and the dark side, too: a review and agenda for research on other-oriented motives, behavior, and impact in organizations. Acad Manag Ann. (2016) 10:1–94. 10.5465/19416520.2016.1153260

[46] Boris Nikolaev Nadav Shir Johan Wiklund. Dispositional positive and negative affect and self-employment transitions: the mediating role of job satisfaction. Entrep Theory Pract. (2019) 44:451–74. 10.1177/1042258718818357

[47] Weiss HM. Deconstructing job satisfaction: separating evaluations, beliefs and affective experiences. Hum Resour Manag Rev. (2003) 12:173–94. 10.1016/S1053-4822(02)00045-1

[48] Orsila R Luukkaala T Manka ML Nygard CHK. A new approach to measuring work-related well-being. Int J Occup Saf Ergon. (2011) 17:341–59. 10.1080/10803548.2011.11076900 22152501

[49] Diener E. Subjective well-being. The Science of Happiness and a Proposal for a National Index. Am Psychol. (2000) 55:34. 10.1037/0003-066X.55.1.34 11392863

[50] Pasamar S Alegre J. Adoption and use of work-life initiatives: looking at the influence of institutional pressures and gender. Eur Manag J. (2015) 33:214–24. 10.1016/j.emj.2014.09.002

[51] Park SH Li S David KT. Market liberalization and firm performance during China's economic transition. J Int Bus Stud. (2006) 37:127–47. 10.1057/palgrave.jibs.8400178

[52] Haveman HA Jia N Shi J Wang Y. The dynamics of political embeddedness in China. Admin Sci Q. (2017) 62:67–104. 10.1177/0001839216657311

[53] Zhou W. Institutional environment, public-private hybrid forms, and entrepreneurial reinvestment in a transition economy. J Bus Ventur. (2017) 32:197–214. 10.1016/j.jbusvent.2016.11.002

[54] Peng Y. Kinship networks and entrepreneurs in China's transitional economy. Am J Sociol. (2004) 109:1045–74. 10.1086/382347

[55] Granovetter M. Economic Action and Social Structure: The Problem of Embeddedness. In: The Sociology of Economic Life. New York, NY: Routledge (2018). p. 22–45. 10.4324/9780429494338-3

[56] Pathak S Muralidharan E. Informal institutions and their comparative influences on social and commercial entrepreneurship: the role of in-group collectivism and interpersonal trust. J Small Bus Manag. (2016) 54:168–88. 10.1111/jsbm.12289

[57] Pagán-Castao E Maseda-Moreno A Santos-Rojo C. Wellbeing in work environments. J Bus Res. (2020) 115. 10.1016/j.jbusres.2019.12.007

[58] Brieger S Clercq DD Meynhardt T. Doing Good, Feeling Good? entrepreneurs' social value creation beliefs and work-related well-being. J Bus Ethics. (2020) 172:707–25. 10.1007/s10551-020-04512-6

[59] Hakanen JJ Schaufeli WB. Do burnout and work engagement predict depressive symptoms and life satisfaction? A three-wave seven-year prospective study. j of Affect Disord. (2012) 141:415–24. 10.1016/j.jad.2012.02.043 22445702

[60] Westhuizen SVD. Work related well-being: burnout, work engagement, occupational stress and job satisfaction within a medical laboratory setting. J Psychol Afr. (2013) 23:467–74. 10.1080/14330237.2013.10820653

[61] Nielsen K Munir F. How do transformational leaders influence followers' affective well-being? Exploring the mediating role of self-efficacy. Work Stress. (2009) 23:313–29. 10.1080/02678370903385106

[62] Schaufeli WB Salanova M. González-romá V, Bakker AB. The measurement of engagement and burnout: a two sample confirmatory factor analytic approach. J Happiness Stud. (2002) 3:71–92. 10.1023/A:1015630930326

[63] Cuyper ND Elst TV Broeck AVD Witte HD. The mediating role of frustration of psychological needs in the relationship between job insecurity and work-related well-being. Work Stress. (2012) 26:252–71. 10.1080/02678373.2012.703900

[64] Boswell WR Boudreau JW Tichy J. The relationship between employee job change and job satisfaction: the honeymoon-hangover effect. J Appl Psychol. (2005) 90:882–92. 10.1037/0021-9010.90.5.882 16162061

[65] Wright TA. The emergence of job satisfaction in organizational behavior: a historical overview of the dawn of job attitude research. J Manag Hist. (2006) 12:262–77. 10.1108/17511340610670179

[66] Warr P. Work, Happiness and Unhappiness. 1st edition. New York, NY: Psychology Press (2011). p. 1-548. 10.4324/9780203936856

[67] Warr P. The study of well-being, behaviour and attitudes. Psychology at Work. (2002).

[68] Ilies R Wilson KS Wagner DT. The spillover of daily job satisfaction onto employees' family lives: the facilitating role of work-family integration. Acad Manag J. (2009) 52:87–102. 10.5465/amj.2009.36461938

[69] Zheng Q Luo Y Wang SL. Moral degradation, business ethics, and corporate social responsibility in a transitional economy. J Bus Ethics. (2014) 120:405–21. 10.1007/s10551-013-1668-4

[70] O'Neil I Ucbasaran D. Balancing “What Matters to Me” with “What Matters to Them”: exploring the legitimation process of environmental entrepreneurs. J Bus Ventur. (2016) 31:133–52. 10.1016/j.jbusvent.2015.12.001

[71] Broeck AVD Ferris DL Chang CH Rosen CC. a review of self-determination theory basic psychological needs at work. J Manag. (2016) 42:1195–29. 10.1177/0149206316632058

[72] Ryan RM Huta V Deci EL. Living well: a self-determination theory perspective on Eudaimonia. J Happiness Stud. (2008) 9:139–70. 10.1007/s10902-006-9023-429524331

[73] Steel RP. Turnover theory at the empirical interface: problems of fit and function. Acad Manag Rev. (2002) 27:346–60. 10.5465/amr.2002.7389900

[74] Judge Weiss HM Kammeyer-Mueller JD Hulin CL. Job attitudes, job satisfaction, and job affect: a century of continuity and of change. J Appl Psychol. (2017) 102:356-74. 10.1037/apl0000181 28125260

[75] Schaumberg RL Flynn FJ. Clarifying the link between job satisfaction and absenteeism: the role of guilt proneness. J Appl Psychol. (2017) 102:982–92. 10.1037/apl0000208 28277721

[76] Wang M Zhan Y Liu S Shultz KS. Antecedents of bridge employment: a longitudinal investigation. J Appl Psychol. (2008) 93:818–30. 10.1037/0021-9010.93.4.818 18642986

[77] Valentine S Godkin L Fleischman GM Kidwell R. Corporate ethical values, group creativity, job satisfaction and turnover intention: the impact of work context on work response. J Bus Ethics. (2011) 98:353–72. 10.1007/s10551-010-0554-6

[78] Grant AM. Leading with meaning: beneficiary contact, prosocial impact, and the performance effects of transformational leadership. Acad Manag J. (2012) 55:458–76. 10.5465/amj.2010.0588

[79] Grant AM Sumanth JJ. Mission Possible? The performance of prosocially motivated employees depends on manager trustworthiness. J Appl Psychol. (2009) 94:927–44. 10.1037/a0014391 19594235

[80] Spector PE Dwyer DJ Jex SM. Relation of job stressors to affective, health, and performance outcomes: a comparison of multiple data sources. J Appl Psychol. (1988) 73:11–9. 10.1037/0021-9010.73.1.11 3350784

[81] Spielberger CD. Anxiety and Behavior. 1st Edition. Cambridge: Academic Press (1966).

[82] McCarthy JM Trougakos JP Cheng BH. Are anxious workers less productive workers? it depends on the quality of social exchange. J Appl Psychol. (2016) 101:279. 10.1037/apl0000044 26375962

[83] Azmat F Ferdous AS Couchman P. Understanding the dynamics between social entrepreneurship and inclusive growth in subsistence marketplaces. J Public Policy Mark. (2015) 34:150702153813007. 10.1509/jppm.14.150

[84] Mair J Marti I. Entrepreneurship in and around institutional voids: a case study from Bangladesh. J Bus Ventur. (2009) 24:419–35. 10.1016/j.jbusvent.2008.04.006

[85] Wang H Alon I Kimble C. Dialogue in the dark: shedding light on the development of social enterprises in China. Glob Bus Organ Excell. (2015) 34:60–9. 10.1002/joe.21615

[86] Townsend DM Hart TA. Perceived institutional ambiguity and the choice of organizational form in social entrepreneurial ventures. Entrep Theory Pract. (2008) 32:685–700. 10.1111/j.1540-6520.2008.00248.x

[87] Gundry LK Kickul JR Griffiths MD Bacq SC. Creating Social Change out of Nothing: The Role of Entrepreneurial Bricolage in Social Entrepreneurs' Catalytic Innovations. Social and Sustainable Entrepreneurship Emerald Group Publishing Limited. (2011). 10.1108/S1074-7540(2011)0000013005

[88] Mair J Marti I Ventresca MJ. Building inclusive markets in rural bangladesh: how intermediaries work institutional voids. Acad Manag J. (2012) 55:819–50. 10.5465/amj.2010.0627

[89] Muñoz P Kibler E. Institutional complexity and social entrepreneurship: a fuzzy-set approach. J Bus Res. (2016) 69:1314–8. 10.1016/j.jbusres.2015.10.098

[90] Nicholls A. Institutionalizing social entrepreneurship in regulatory space: reporting and disclosure by community interest companies. Account Organ Soc. (2010) 35:394–415. 10.1016/j.aos.2009.08.001

[91] Beutell NJ Alstete JW Schneer JA Hutt C. A Look at the dynamics of personal growth and self-employment exit. Int J Entrepreneurial Behave Res. (2019) 25:1452–70. 10.1108/IJEBR-04-2018-0239

[92] Hoogendoorn B Zwan PVD Thurik R. Sustainable entrepreneurship: the role of perceived barriers and risk. J Bus Ethics (2019) 157:1133–54. 10.1007/s10551-017-3646-8

[93] Cacciotti G Hayton JC Mitchell JR Allen DG. Entrepreneurial fear of failure: scale development and validation. J Bus Ventur. (2020) 35:106041. 10.1016/j.jbusvent.2020.106041

[94] Thompson NA Gelderen MV Keppler L. No Need to Worry? Anxiety and coping in the entrepreneurship process. Front Psychol. (2020) 11:398. 10.3389/fpsyg.2020.00398 32226405 PMC7080856

[95] Calderwood C Bennett AA Gabriel AS Trougakos JP Dahling JJ. Too anxious to help? Off-job affective rumination as a linking mechanism between work anxiety and helping. J Occup Organ Psychol. (2018) 91:681–7. 10.1111/joop.12220

[96] Cropley M Zijlstra FR. Work and Rumination. Handbook of stress in the occupations. (2011) 487:503. 10.4337/9780857931153.00061

[97] Vahle-Hinz T Mauno S De Bloom J Kinnunen U. Rumination for innovation? Analysing the longitudinal effects of work-related rumination on creativity at work and off-job recovery. Work Stress. (2017) 31:315–37. 10.1080/02678373.2017.1303761

[98] Bakker AB Schaufeli WB Sixma HJ Bosveld W. Burnout contagion among general practitioners. J Soc Clin Psychol. (2001) 20:82–98. 10.1521/jscp.20.1.82.22251

[99] Li S Song X Wu H. Political connection, ownership structure, and corporate philanthropy in China: a strategic-political perspective. J Bus Ethics. (2015) 129:399–411. 10.1007/s10551-014-2167-y

[100] Cherrier H Goswami P Ray S. Social entrepreneurship: creating value in the context of institutional complexity. J Bus Res. (2018) 86:245–58. 10.1016/j.jbusres.2017.10.056

[101] Chatterjee I Cornelissen J Wincent J. Social entrepreneurship and values work: the role of practices in shaping values and negotiating change. J Bus Ventur. (2021) 36:106064. 10.1016/j.jbusvent.2020.106064

[102] De Bakker FG Den Hond F King B Weber K. Social movements, civil society and corporations: taking stock and looking ahead. Organ Stud. (2013) 34:573–93. 10.1177/0170840613479222

[103] Baumeister RF Vohs KD Tice DM. The strength model of self-control. Curr Dir Psychol Sci. (2007) 16:351–5. 10.1111/j.1467-8721.2007.00534.x

[104] Tice D Baumeister R Shmueli Blumberg D Muraven M. Restoring the self: Positive affect helps improve self-regulation following ego-depletion. J Exp Soc Psychol. (2007) 43:379–84. 10.1016/j.jesp.2006.05.007

[105] Kibler E Wincent J Kautonen T Cacciotti G Obschonka M. Can prosocial motivation harm entrepreneurs' subjective well-being? J Bus Ventur. (2019) 34:608–24. 10.1016/j.jbusvent.2018.10.003

[106] Leiter MP Maslach C. The impact of interpersonal environment on burnout and organizational commitment. J Organ Behav. (1988) 9:297–308. 10.1002/job.4030090402

[107] Jex SM. Stress and Job Performance: Theory, Research, and Implications for Managerial Practice. New York, NY: Sage Publications Ltd (1998).

[108] Schaufeli WB Bakker AB. Job demands, job resources, and their relationship with burnout and engagement: a multi-sample study. J Organ Behav. (2004) 25:293–315. 10.1002/job.248

[109] Sardeshmukh SR Goldsby M Smith RM. Are work stressors and emotional exhaustion driving exit intentions among business owners? J Small Bus Manag. (2018) 59:544–74. 10.1111/jsbm.12477

[110] Puffer SM Mccarthy DJ Boisot M. Entrepreneurship in Russia and China: the impact of formal institutional voids. Entrep Theory Pract. (2010) 34:441–67. 10.1111/j.1540-6520.2009.00353.x

[111] Nicholls A. The legitimacy of social entrepreneurship: Reflexive isomorphism in a pre-paradigmatic field. Entrep Theory Pract. (2010) 34:611–33. 10.1111/j.1540-6520.2010.00397.x

[112] Shepherd DA McMullen JS Jennings PD. The formation of opportunity beliefs: overcoming ignorance and reducing doubt. Strateg Entrepreneurship J. (2007) 1:75–95. 10.1002/sej.3

[113] Partzsch L Ziegler R. Social entrepreneurs as change agents: a case study on power and authority in the water sector. Int Environ Agreements. (2011) 11:63–83. 10.1007/s10784-011-9150-1

[114] Letaifa SB. How social entrepreneurship emerges, develops and internationalises during political and economic transitions. Eur J Int Manag. (2016) 10:455–66. 10.1504/EJIM.2016.077424

[115] Dey P Schneider H Maier F. Intermediary organisations and the hegemonisation of social entrepreneurship: fantasmatic articulations, constitutive quiescences, and moments of indeterminacy. J Organ Stud. (2016) 37:1451–72. 10.1177/0170840616634133

[116] Tiwari P Bhat AK Tikoria J. Factors Affecting Individual's Intention to Become a Social Entrepreneur. In: Agrawal A Kumar P editors. Social Entrepreneurship and Sustainable Business Models: The Case of India. Cham: Springer International Publishing (2018). p. 59-98. 10.1007/978-3-319-74488-9_3

[117] Dickson BJ. Red Capitalists in China: The Party, Private Entrepreneurs, and Prospects for Political Change. Cambridge: Cambridge University Press (2003). 10.1017/CBO9780511510045

[118] Aronson E Wilson TD Akert RM. Social Psychology: Media and Research Update. 4th ed. New Jersey: Pearson Prentice Hall (2004).

[119] Brislin RW. Translation and Content Analysis of Oral and Written Materials. Methodology (1980):389–444.

[120] Nunnally JC. Psychometric Theory 3e. Tata McGraw-hill education (1994).

[121] Lepoutre J Justo R Terjesen S Bosma N. Designing a global standardized methodology for measuring social entrepreneurship activity: the global entrepreneurship monitor social entrepreneurship study. Small Bus Econ. (2013) 40:693–714. 10.1007/s11187-011-9398-4

[122] Estrin S Mickiewicz T Stephan U. Entrepreneurship, social capital, and institutions: social and commercial entrepreneurship across nations. Entrep Theory and Pract. (2013) 37:479–504. 10.1111/etap.12019

[123] Pollack JM Vanepps EM Hayes AF. The moderating role of social ties on entrepreneurs' depressed affect and withdrawal intentions in response to economic stress. J Organ Behav. (2012) 33:789–810. 10.1002/job.1794

[124] Grant A. Does intrinsic motivation fuel the prosocial fire? motivational synergy in predicting persistence, performance, and productivity. J Appl Psychol. (2008) 93:48–58. 10.1037/0021-9010.93.1.48 18211134

[125] Scarpello V Campbell JP. Job satisfaction: are all the parts there? Pers Psychol. (1983) 36:577–600. 10.1111/j.1744-6570.1983.tb02236.x

[126] Moynihan DP Pandey SK. Finding workable levers over work motivation: comparing job satisfaction, job involvement, and organizational commitment. Adm Soc. (2007) 39:803–32. 10.1177/0095399707305546

[127] Chordiya R Sabharwal M Goodman D. Affective organizational commitment and job satisfaction: a cross-national comparative study. Public Adm. (2017) 95:178–95. 10.1111/padm.12306

[128] Haider S Fatima N Pablos-Heredero CD. A Three-Wave Longitudinal Study of Moderated Mediation between Perceptions of Politics and Employee Turnover Intentions: The Role of Job Anxiety and Political Skills. Revistade Psicologiadel Trabajoydelas Organizaciones. (2020) 36:1–14. 10.5093/jwop2020a1

[129] Malach-Pines Ayala. The burnout measure, short version. Int J Stress Manag. (2005) 12:78-88. 10.1037/1072-5245.12.1.78

[130] Li H Meng L Wang Q Zhou L-A. Political connections, financing and firm performance: evidence from chinese private firms. J Dev Econ. (2008) 87:283–99. 10.1016/j.jdeveco.2007.03.001

[131] Wang H Qian C. Corporate philanthropy and corporate financial performance: the roles of stakeholder response and political access. Acad Manag J. (2011) 54:1159–81. 10.5465/amj.2009.0548

[132] Jia N. Are collective political actions and private political actions substitutes or complements? Empirical evidence from China's private sector. Strateg Manag J. (2014) 35:292–315. 10.1002/smj.2092

[133] Ma D Parish WL. Tocquevillian moments: charitable contributions by Chinese private entrepreneurs. Social Forces. (2006) 85:943–64. 10.1353/sof.2007.0016

[134] Mayr U Freund AM. do we become more prosocial as we age, and if so, why? Curr Dir Psychol Sci. (2020) 29:248–54. 10.1177/096372142091081130710411

[135] Harding R. Global Entrepreneurship Monitor: United Kingdom 2004. Rochester, NY: Social Science Electronic Publishing. (2006) 3:66–70.

[136] Justo R DeTienne DR Sieger P. Failure or voluntary exit? reassessing the female underperformance hypothesis. J Bus Ventur. (2015) 30:775–92. 10.1016/j.jbusvent.2015.04.004

[137] Lin S Wang S. How does the age of serial entrepreneurs influence their re-venture speed after a business failure? Small Bus Econ. (2019) 52:651–66. 10.1007/s11187-017-9977-0

[138] Gefen D Rigdon EE Straub D. Editor's Comments: An Update and Extension to Sem Guidelines for Administrative and Social Science Research. Mis Quart. (2011) 35:1–7. 10.2307/23044042

[139] Chin WW Newsted PR. Structural equation modeling analysis with small samples using partial least squares. Statistical Strategies for Small Sample Research. (1999) 1:307–41.31504624

[140] Anderson JC Gerbing DW. Structural equation modeling in practice: a review and recommended two-step approach. Psychol Bull. (1988) 103:411. 10.1037/0033-2909.103.3.411

[141] Hair JF Jr Hult GTM Ringle CM Sarstedt M. A Primer on Partial Least Squares Structural Equation Modeling (Pls-Sem. London: Sage publications (2021). 10.1007/978-3-030-80519-7

[142] Fornell C Larcker DF. Evaluating structural equation models with unobservable variables and measurement error. J Mark Res. (1981) 18:39–50. 10.1177/002224378101800313

[143] Gold AH Malhotra A Segars AH. Knowledge management: an organizational capabilities perspective. Manag Inf Syst. (2001) 18:185–214. 10.1080/07421222.2001.11045669

[144] Hair JF Jr Sarstedt M Ringle CM Gudergan SP. Advanced Issues in Partial Least Squares Structural Equation Modeling. London: sage publications (2017). 10.1007/978-3-319-05542-8_15-1

[145] Sobel ME. Asymptotic confidence intervals for indirect effects in structural equation models. Sociol Methodol. (1982) 13:290–312. 10.2307/270723

[146] Lee C Hallak R. Investigating the moderating role of education on a structural model of restaurant performance using multi-group pls-sem analysis. J Bus Res. (2018) 88:298–305. 10.1016/j.jbusres.2017.12.004

[147] Spivack AJ McKelvie A Haynie JM. Habitual entrepreneurs: possible cases of entrepreneurship addiction? J Bus Ventur. (2014) 29:651–67. 10.1016/j.jbusvent.2013.11.002

[148] Inglehart R. Mapping global values. Comp Sociol. (2006) 5:115–36. 10.1163/156913306778667401

[149] Diener E Scollon CN Lucas RE. The evolving concept of subjective well-being: the multifaceted nature of happiness. Springer Netherlands. (2009). 10.1007/978-90-481-2354-4_4

[150] Hsu DK Wiklund J Anderson SE Coffey BS. Entrepreneurial exit intentions and the business-family interface. J Bus Ventur. (2016) 31:613–27. 10.1016/j.jbusvent.2016.08.001

[151] Zhu F Burmeister-Lamp K Hsu DK. To Leave or Not? The impact of family support and cognitive appraisals on venture exit intention. Int J Entrepreneurial Behav Res. (2017) 23:566–90. 10.1108/IJEBR-04-2016-0110

[152] Rauch A Frese M. Psychological approaches to entrepreneurial success: a general model and an overview of findings. Organ Psychol. (2000) 15.

[153] Zhou W. Bank financing in China's Private Sector: the Payoffs of political capital. World Dev. (2009) 37:787–99. 10.1016/j.worlddev.2008.07.011

[154] Parker SC. The Economics of Entrepreneurship: Cambridge University Press (2018).

[155] Purvanova RK Muros JP. gender differences in burnout: a meta-analysis. J Vocat Behav. (2010) 77:168–85. 10.1016/j.jvb.2010.04.006

[156] Sastre-Castillo MA Peris-Ortiz M Danvila-Del Valle I. What is different about the profile of the social entrepreneur? Nonprofit Manag Leadersh. (2015) 25:349–69. 10.1002/nml.2113823156922

[157] Dickel P Eckardt G. Who wants to be a social entrepreneur? The role of gender and sustainability orientation. J Small Bus Manag. (2021) 59:196–218. 10.1080/00472778.2019.1704489

[158] Smith DM Langa KM Kabeto MU Ubel PA. Health, wealth, and happiness: financial resources buffer subjective well-being after the onset of a disability. Psychol Sci. (2005) 16:663–6. 10.1111/j.1467-9280.2005.01592.x 16137249

[159] Grover S Helliwell JF. How's Life at Home? New Evidence on Marriage and the Set Point for Happiness. J Happiness Stud. (2019) 20:373–90. 10.1007/s10902-017-9941-3

[160] DeTienne DR Cardon MS. Impact of Founder Experience on Exit Intentions. Small Bus Econ. (2012) 38:351–74. 10.1007/s11187-010-9284-5

[161] Bacq S Janssen F. The multiple faces of social entrepreneurship: a review of definitional issues based on geographical and thematic criteria. Entrepreneurship Reg Dev. (2011) 23:373–403. 10.1080/08985626.2011.577242

